# Shortening Tuberculosis treatment schedules in children

**DOI:** 10.1038/s43856-022-00109-4

**Published:** 2022-04-07

**Authors:** Katharine Barnes

**Affiliations:** Communications Medicine, https://www.nature.com/commsmed

## Abstract

Most clinical trials to determine optimal tuberculosis (TB) treatment regimens have been carried out in adults. A recent randomised control clinical trial published in *The New England Journal of Medicine* compared the response in children following 4 or 6 months of standard first-line anti-TB treatment and found it to be similar.

TB in children is often less severe than that seen in adults. The presence of disease in children can be hard to confirm as often the presence of bacteria in respiratory samples is not visible under the microscope. Nevertheless, treatment is recommended in suspected cases to prevent disease progression and spread. Differences in pharmacokinetics following TB treatment have also been seen between adults and children. Recently, it has been shown that TB treatment times can be shortened for adults with mild disease. However, given the many differences between adults and children with TB, it could not be assumed the same would apply to children.

**Figure:**
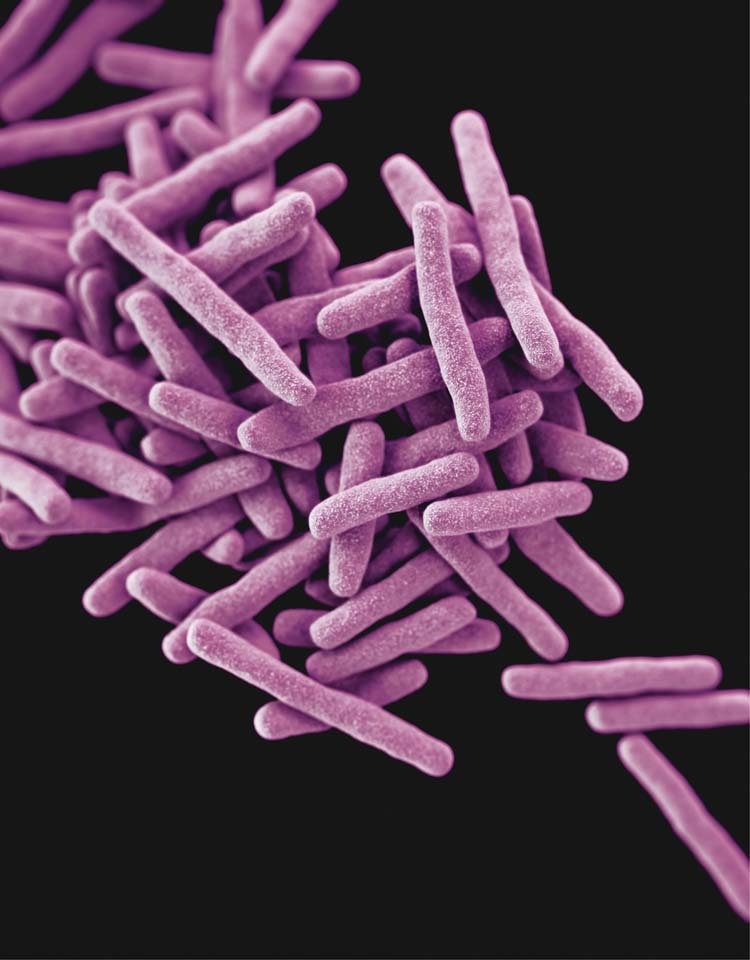
Photo by CDC on Unsplash

Thus, Turkova et al. undertook a treatment-shortening trial on 1204 children thought to be drug-susceptible with non-severe, symptomatic, smear-negative tuberculosis and the results have recently been published in *The New England Journal of Medicine*^1^. The trial was undertaken in Uganda, Zambia, South Africa and India, countries that have high TB prevalence. All trial participants initially received 8 weeks of standard intensive therapy, comprising isoniazid, rifampin, and pyrazinamide, with or without ethambutol according to local guidelines. This was followed with either 8 or 16 weeks of isoniazid and rifampin.

The primary efficacy outcome measured was unfavourable status by 72 weeks following the start of treatment. Unfavourable status was classified as loss to follow up, death or tuberculosis events, which comprised treatment failure, treatment extension, anti-tuberculosis-treatment drug change, treatment restart due to suspected treatment failure, and tuberculosis recurrence. Adverse events were also assessed, as the primary safety outcome. Secondary endpoints were unfavourable status in those participants confirmed to have had TB at the start, death, adverse drug reactions, bacterial infection leading to hospitalisation, adherence to the treatment regimen, and acceptability of treatment as determined by the caregiver or child. For all these endpoints, no statistically significant differences were seen between both groups.

In addition, economic analyses were carried out to estimate costs and health outcomes in terms of life-years and quality-adjusted life-years. After 72 weeks’ participants who had been treated for 4 months had similar health outcomes to those who had been treated for 6 months, but with lower health care costs.

The results of this trial demonstrate that shortening TB treatment for children with mild TB does not compromise treatment effectiveness, but has positive consequences due to the reduced cost and reduced time spent in treatment. As a consequence, the World Health Organisation has changed the global guidelines for managing TB in children. Given that two thirds of children with TB do not have severe disease, the majority of children with TB will benefit from this change in the guidelines.
